# Generation of highly realistic microstructural images of alloys from limited data with a style-based generative adversarial network

**DOI:** 10.1038/s41598-023-27574-8

**Published:** 2023-01-11

**Authors:** Guillaume Lambard, Kazuhiko Yamazaki, Masahiko Demura

**Affiliations:** 1 National Institute for Materials Science (NIMS), Research and Services Division of Materials Data and Integrated System (MaDIS), 305-0044 Namiki 1-1, Tsukuba, Ibaraki Japan; 2 JFE Steel Corporation, Steel Research Laboratory, 210-0855 1-1 Minamiwataridacho, Kawasaki, Kanagawa Japan

**Keywords:** Materials science, Metals and alloys

## Abstract

In materials science, the amount of observational data is often limited by operating protocols that require a high level of expertise, often machine-dependent, developed for a time-consuming integration of valuable data. Scanning electron microscopy (SEM) is one of those methodologies of characterisation for which the number of observations of a given material is limited to just a few images. In the present study, we present the possibility to artificially inflate the size of SEM image datasets from a limited ($\mathrm \sim 100s-1000s$ of images) to a virtually unbounded number thanks to a generative adversarial network (GAN). For this purpose, we use one of the latest developments in GAN architectures and training methodologies, the StyleGAN2 with adaptive discriminator augmentation (ADA), to generate a diversity of high-quality SEM images of $\mathrm 512\times 512$ pixels. Overall, coarse and fine microstructural details are successfully reproduced when training a StyleGAN2 with ADA from scratch on at most 3000 SEM images, and interpolations between microstructures are performed without significant modifications to the training protocol when applied to natural images.

## Introduction

The recent advent of generative adversarial networks (GAN)^1–7^ in the creation of high-resolution synthetic images, indistinguishable from real counterparts, has triggered a revolution in various domains of graphical contents creation. However, the exploitation of this ability had been limited to applications with access to an ample supply of images ($\mathrm \sim 10^5 - 10^6$) until recently. The adaptive discriminator augmentation (ADA) mechanism proposed in Karras et al.^8^ significantly stabilises the training of a GAN in a limited data regime, avoiding the discriminator, the neural network intended to classify images as real or generated, to overfit on training data^9,10^. More specifically, the style-based GAN architecture (StyleGAN2)^11^, borrowed from style transfer literature^12^, with ADA yields state-of-the-art results in data-driven unconditional generative image modelling, both in terms of existing distribution quality metrics as well as perceived image quality in low data regime ($\sim 10^3 - 10^4$)^8^. This decrease by two orders of magnitude of the number of images necessary to train a GAN for generating highly realistic images significantly reduces the gap for applying a GAN to domains where the collection of a large enough set of images is time-consuming, costly, or remains constrained by the limited quantity of a material to be characterised, such as in medicine^13^, or materials science^14^.

The present study focuses explicitly on images acquired by scanning electron microscopy (SEM). A sub-field of electron microscopy subjects to a lack of available data, and for which the accumulation of diverse high-quality images demands expertise in preparing a sample, calibrating an SEM apparatus, finding areas of observational interest, and acquiring high-quality images. The quality (e.g., contrast, sharpness, noise, aliasing) and diversity (e.g., shape, fraction, and orientation of grains, grain boundary aspects, fine microstructural details of phases) of a set of SEM images depend on the operator’s abilities as well as on the microstructure intrinsic to a sample, which often constrains to require multiple attempts before insightful SEM images are obtained. Therefore, acquired limited data can only capture some of the edge cases existing in the space of microstructures attainable from the synthesis and process of a given alloy. In this sense, the programmatic generation of synthetic SEM data allows observational prospects on many microstructural scenarios from slightly (e.g., the spatial distribution of phases) to highly (e.g., interpolation, or mixture, between two different microstructures) perturbed by comparison to the training set of real SEM images.

In the present manuscript, alloys of ferrite-martensite dual-phase (DP) steel are the main focus of observations via SEM. DP steels include a soft ferrite matrix containing islands of hard martensite. They are suggested to be good candidates for the next generation of steels for the automotive industry^15^ where steel materials with high tensile strength (TS) and high elongation at failure (EL) are necessary to achieve both weight reduction and collision safety. Numerous studies have pushed forward the capabilities of DP steels for more than 50 years^16^. However, the strength-ductility trade-off, i.e., an increase in TS generally implies a decrease in EL, is still to be overcome. The tensile properties of DP steels are expected to depend on the spatial arrangement and specific internal structure of ferrite and martensite phases. Experimental studies have shown that TS is improved in a chain-like networked DP structure^17^ or by increasing the martensite volume fraction and grain refinement^18,19^. The martensite grain width is also reported to affect EL by combining in-situ observations and finite element methods (FEM)^20^. The appearance, growth, and merging of voids that affect local deformations in alloys depend on the microstructure, and FEM that considers strain localization due to microstructure with various fracture mechanisms are desirable to predict local EL^21^. Additionally, FEM highlights that a more significant aspect ratio in martensite grains provides a more uniform elongation^22^. Nevertheless, the diverse relationships between microstructural features and the product $\mathrm{TS} \times \mathrm{EL}$ are not yet sorted out and known^23^.

Meanwhile, deep learning methodologies offer new perspectives to the electron microscopy field for most of the potential applications like compressed sensing, semantic segmentation, labelling, deblurring, enhanced image resolution^24^, or prediction of alloys property and in inverse design. For example, Ishiyama et al.^25^ show the importance of introducing a large set of synthetic images to increase the performance of a convolution neural network trained to predict the diameter and density of target microstructural features directly from images. Indeed, the synthesis of under-represented data can be a remedy to support the decision-making process issued from imbalanced datasets^26^. Overall, SEM data can serve classification or regression tasks through supervised learning, i.e., supported by manually labelled SEM images, to identify phases from which the extraction of microstructural features can serve the prediction of mechanical properties^27^, or to build a microstructure-property relationship directly from SEM images to target properties^28^, where manual phases segmentation and microstructural features extraction are both unnecessary. Among the non-exhaustive usages of deep learning in the electron microscopy field mentioned above, all could benefit from the generation of realistic SEM image populations for emancipating their generalization to experimentally unobserved, nevertheless observable microstructural features and for improving their respective performance. In this sense, generative models like a StyleGAN2 with ADA are intended to create synthetic yet realistic populations of SEM images to serve image-based deep learning models intended to classify, segment, or characterize microstructures, as well as FEM simulations in which fine microstructural features of phases, such as in martensite islands in DP steels, should prove to be of utility in disentangling the strength-ductility trade-off.

To this end, a methodology based on a StyleGAN2 with ADA dedicated to low data regimes allows enriching pools of SEM images that fit a proper joint distribution of microstructural characteristics. It is worth noting that the space of microstructural characteristics is expected to be high-dimensional in the sense that coarse (e.g., phases spatial arrangement) and fine (e.g., the internal structure of the martensite phase) observable details, as well as more complex distinguishable patterns, are all intended to be captured by the generative model with the advantage to synthesize realistic SEM images yet different from the original data. Furthermore, this approach is expected to support the diversity of SEM image populations in improving the frequency of under-represented microstructural characteristics, which may have an otherwise difficultly noticeable impact on overall microstructures and related properties. The current pipeline using a StyleGAN2 with ADA should adequately serve this purpose thanks to the expected high quality of generated SEM images and respect for the joint distribution of DP steel alloy microstructural features.

Here, we use a private dataset of 3000 SEM images of ferrite-martensite DP steel sheets acquired at JFE Steel Corporation. To this end, 30 ferrite-martensite DP steel sheets were prepared with a martensite fraction ranging from 37% to 100%. The protocols of synthesis and observation of the ferrite-martensite DP steel sheets are detailed in Methods. A StyleGAN2 architecture is trained with success following a slightly tailored ADA protocol on our dataset, by comparison to the nominal protocol introduced in Karras et al.^8^, and is later used as an unconditional generative model of realistic $\mathrm 512 \times 512$ SEM images. The pre-processing of SEM images, general settings for a training of a StyleGAN2 with ADA, and fixed hyper-parameters are indicated in Methods. In the Results, we detail the impact on the quality of output SEM images of the ADA protocol (i.e., type of image augmentations, frequency of adaptation to over-fitting) relevant to a dataset of SEM images, as well as the dependency of the diversity in generated SEM images on the size of the training set. In other words, we propose a primed ADA protocol and a lower bound on the size of a training set for a StyleGAN2-like GAN architecture to successfully extract and combine individual modes of structural characteristics from SEM images. Additionally, we demonstrate an ability to produce a smooth interpolation between distinctive microstructures by navigating the latent representation learned by the StyleGAN2 with ADA, resulting in convincing generated $\mathrm 512 \times 512$ SEM images with cross-microstructural features. Finally, we discuss the expected consequences of introducing generative models in observational materials science and potential extensions from this study.

## Results

Following the methodology described in Karras et al.^8^, there exists a large set of image transformations and training hyper-parameters of a StyleGAN2 that may influence the quality and diversity of generated SEM images. Furthermore, the size and the nature (e.g., color scale, coarse and fine details of grains, diversity and type of microstructures) of a training set of SEM images influence the best attainable generative performance. Therefore, we investigate hereafter the variations in terms of quality and diversity of generated SEM images, quantified through the computation of the Fréchet inception distance (FID) and recall metric (see Methods), and settle on a generative pipeline suited for SEM images of alloy microstructures in low data regime.

### Which augmentations for the generation of SEM images?

The dimensionality of 2D images (height, width, and single (L) or three (RGB) channels pixel colors) allows applying a large set of distinct combinations of different types of transformation, such as pixel blitting, geometrical and colors manipulations, image-space filtering, additive noise^29^, and cutout^30^. This manuscript uses the terms transformation and augmentation of images interchangeably. Table 1 in Supplementary Materials provides a random selection of five samples of real greyscale SEM images issued from our dataset, to which those different transformations are applied. However, not all of the listed transformations are meaningful to SEM images. As a general principle, meaningful and purposeful image transformations should be tailored for each dataset of images at hand. Even though some transformations can be quickly dropped based on operating and general SEM image aspect considerations, we first evaluate their individual effectiveness in improving the training performance of the StyleGAN2 with ADA on our dataset of SEM images.

**Figure 1:**
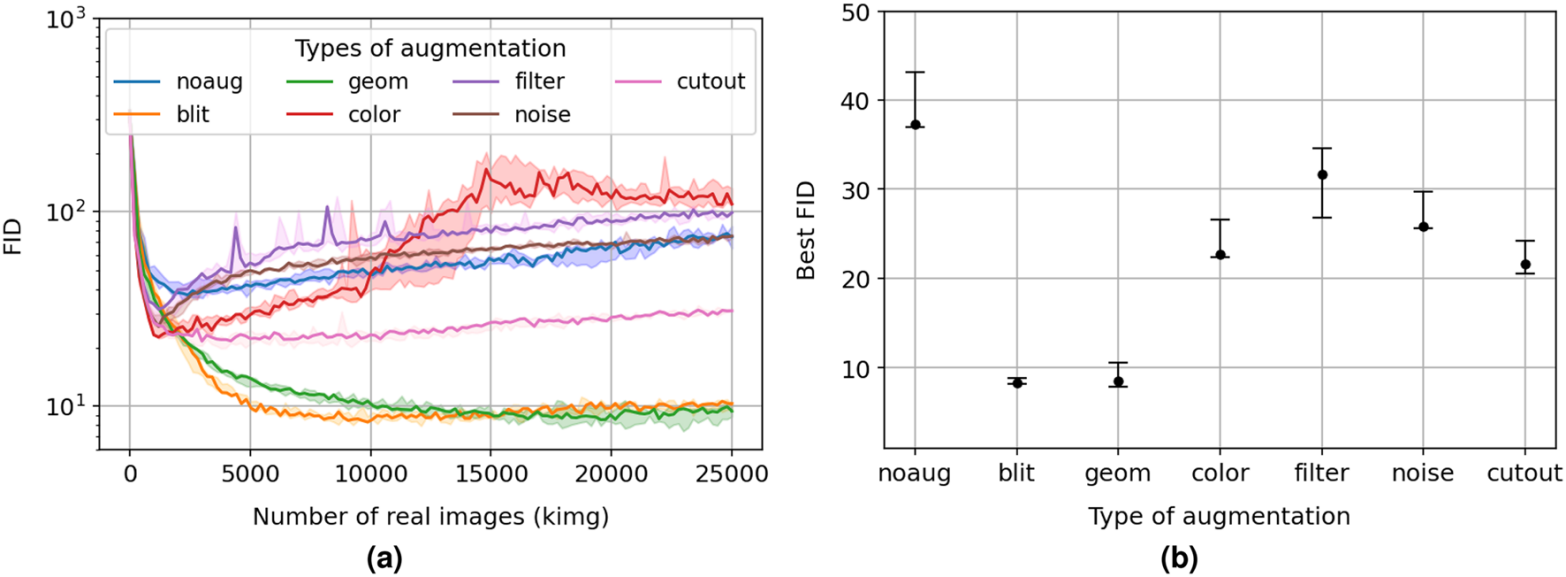
(**a**) Fréchet inception distance (FID, lower is better) of generated to real SEM images as a function of the number of real images (per thousand images, kimg) presented to the discriminator during training of the StyleGAN2 with different types of applied augmentation: no augmentation (noaug in blue), pixel blitting (blit in orange), geometrical (geom in green), color (color in red), image-space filtering (filter in violet), additive noise (noise in brown), and cutout (cutout in pink). For any distribution, the minimum, median, and maximum FID values issued from three independent runs are reported; (**b**) Corresponding median (black dot), minimum (lower cap), and maximum (higher cap) values for the best FID are shown for the different types of augmentation.

Figure 1a shows the impact of each type of augmentation applied to SEM images on the convergence of the FID along the training process of the StyleGAN2, i.e. as a function of the number of SEM images (per thousand of images, kimg) shown to the discriminator. Overall, three independent runs are performed for each type of augmentation (no augmentation included) with default hyper-parameters^31^, i.e. including that all 3000 available SEM images are used for training and the target heuristic $\mathrm r_t = 0.6$^8^ (see below). Image-space filtering (violet), additive noise (brown), and a color-based transformation (red) are among applied augmentations that engender lower FID in the early stage of the training ($\mathrm \sim 1000$ kimg) in comparison to no augmentation at all (blue) by $\mathrm \sim 14,\,30$, and $\mathrm 36$%, respectively (see Fig. 1b). Nevertheless, they are then rapidly subject to over-fitting, as exhibited by a quick rise of the FID. In their case, the ADA protocol is of no help in reducing the over-confidence of the discriminator with nearly an order of magnitude difference in FID with best-performing augmentation strategies by the end of the training process at 25000 kimg. The color-based set {constrast, brightness} of transformations is especially subject to strong instabilities in the training process, even though the Luma flip, i.e. inversion of the luminance, hue rotation and saturation, are intentionally removed for reasons exposed in the Supplementary Materials. This is a behaviour which has already been observed for natural images (e.g., human faces) in Karras et al.^8^, where the probability $\mathrm p$ to perform a color-based transformation must be kept low ($\mathrm p \in [0.2,0.4]$ in Fig. 4b,c in Karras et al.^8^) for a best FID. Consequently, the target heuristic $\mathrm r_t$ must be kept higher than 0.6 (the threshold used here) to avoid a quick rise of $\mathrm p$ in an attempt to decrease over-fitting. More generally and apart from the empirical technicalities linked to the ADA protocol, SEM images used for the training possess *a priori* consistent brightness and contrast among different samples of alloy and human operators, as guaranteed by a pre-processing (see Methods). This last renders obsolete transformations in contrast and brightness as transformed SEM images tend not to represent the dataset at hand and therefore put an intrinsic limitation on the possible improvement of the FID. On par with the color-based transformations, the image-space filtering and additive noise demonstrates a short improvement in the FID with increased stability along the training process. However, real SEM images (see Fig. 3 in Supplementary Materials) show fine textural details characterised by high (sharp edges, thin and elongated structures, rate of grain boundaries) rather than low (large smooth coarse grain with uniform color) frequencies, the decomposition in 4 large bands of frequencies (from 0 to $\mathrm \pi$) produce SEM images that are irrelevant to the dataset at hand. More trivially, the same goes for the additive noise with an FID distribution consistent with no augmentation.

It is worth noting that the cutout (pink in Fig. 1a) decreases the FID with no augmentation by $\mathrm \sim 40$%, an effect not seen on natural images (see Fig. 4a–b in Karras et al.^8^). The cutout transformation used here corresponds to setting pixels to zero within a rectangular area of size $\mathrm{ (width/2, height/2)}$ and centre selected from a uniform distribution over an entire image^30^. Therefore, the cutout, or added mean area in the case that pixel values are normalised on [-1,1], can be treated as a meaningful adversarial attack on SEM images in the form of a coarse grain placement in a microstructure. However, and despite the appeal of the cutout corruption of SEM images, the consequent best FID (see Fig. 1b) remains high by a factor $\mathrm >2$ when compared to the best two performing approaches, pixel blitting and geometrical transformations, with a value relatively close to the one obtained from early convergence with a color-based transformation.

Pixel blitting and geometrical transformations (orange and green in Fig. 1a, respectively), both most used in the deep learning literature for their simplicity of implementation and increase in performance, remain the choice by excellence for natural (see Fig. 4a–b in Karras et al.^8^) as well as SEM images. X-flip, $\mathrm 90^\circ$/arbitrary rotation, integer/fractional translation, and isotropic/anisotropic scaling are among the included transformations that are relevant to the field of SEM microscopy. They assure relaxation on the placement, orientation, and size of the grains and their fine detailed features without disruption of their initial distribution as shown quantitatively by their FID approaching $\mathrm \sim 8$ at best in Fig. 1b, a $\mathrm \sim 77$% improvement to no augmentation.

In consequence, and for the rest of this study, we keep the pixel blitting and geometrical transformations as the two sole augmentation strategies for their simplicity, stable response to the ADA protocol, training efficiency, and relevancy to the domain of SEM images.

### Influence of the target heuristic $\mathrm r_t$

Among the whole set of hyper-parameters that may impact the performance of the StyleGAN2, the target heuristic $\mathrm r_t$ (see Equation 4 in Methods) depends on the dataset, as mentioned in Karras et al.^8^, and deserves to be tuned in the present study. To this end, we train the StyleGAN2 using the pixel blitting and geometrical transformations set above over 25000 kimg and fix the target heuristic $\mathrm r_t \in \left[ 0.3,\,0.9\right]$ with a resolution of $\mathrm 0.1$. As above, three independent runs are performed for a given $\mathrm r_t$ value, each defined with an individual random seed.

**Figure 2:**
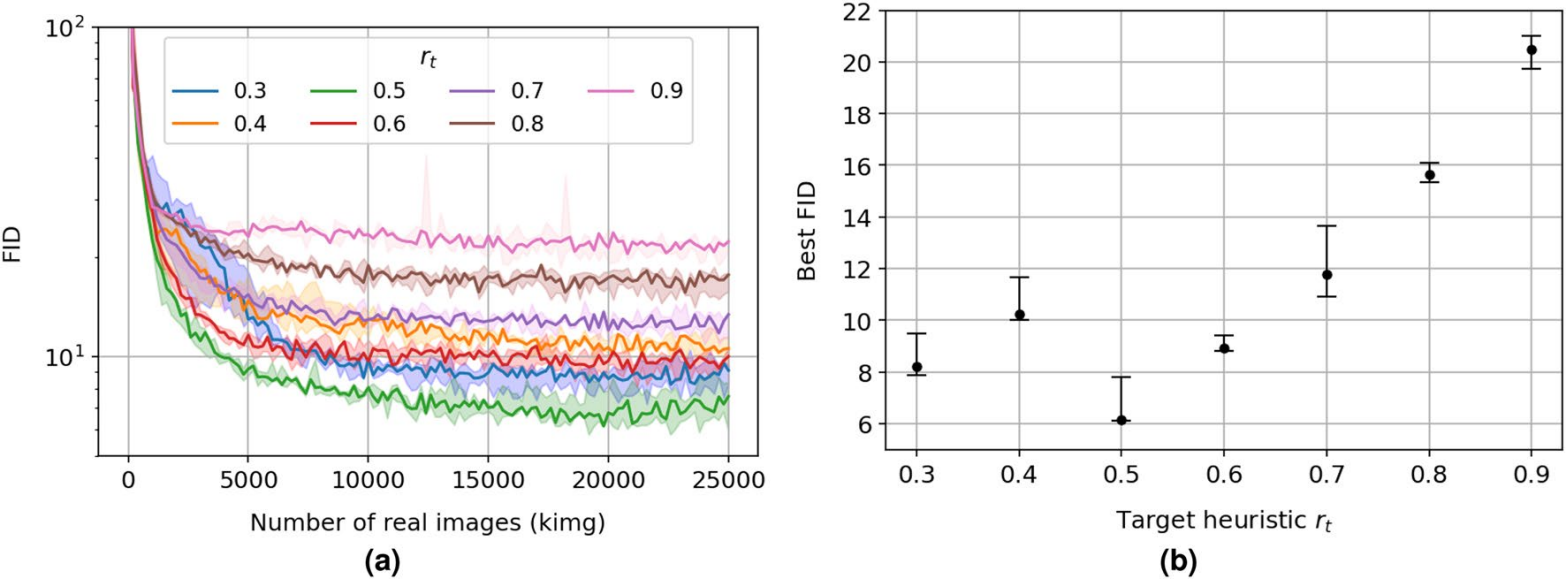
(**a**) Fréchet inception distance (FID, lower is better) of generated to real SEM images as a function of the number of real images (per thousand images, kimg) seen by the discriminator during the training of the StyleGAN2 for different heuristic $\mathrm r_t \in \left\{ 0.3,\,0.4,\,0.5,\,0.6,\,0.7,\,0.8,\,0.9\right\}$ (blue, orange, green, red, violet, brown, and pink, respectively). For any distribution, the minimum, median, and maximum FID values issued from three independent runs are reported; (**b**) Corresponding median (black dot), minimum (lower cap), and maximum (higher cap) values for the best FID are shown for the different fixed $\mathrm r_t$ values.

Figure 2a shows the distributions of the FID for the different fixed $\mathrm r_t$ values along the training process. First of all, it is worth noting that even though statistical instabilities during the training are at most encountered with $\mathrm r_t = 0.3$ under $\mathrm \sim 5000$ kimg, it still reaches convergence with a second-best FID by the end of the training process. Otherwise, FID distributions with $\mathrm r_t > 0.3$ follow a more stable training history (i.e. minimum and maximum FID values tend to be close). Figure 2b illustrates the evolution of the best FID for the different fixed target heuristic $\mathrm r_t$. It displays a tendency of the best FID with $\mathrm r_t$ that is observed as well on images issued from the FFHQ (i.e. human faces) dataset (see Fig. 5b in Karras et al.^8^), when the size of the training set is $\mathrm \le 10$k images. On the other hand, target heuristic $\mathrm r_t \le 0.6$ values tend to bring a more significant improvement on the FID, with the best FID worsening as the $\mathrm r_t$ increases above $\mathrm 0.6$. Interestingly, $\mathrm r_t = 0.5$ brings about the most significant improvement for the FID on SEM and FFHQ images in a low data regime ($\mathrm \le 10$k images). However, a value of $\mathrm 0.6$, more biased towards more significant data regime ($\mathrm \ge 50$k images), is kept as the choice by default in Karras et al.^8^. Here, we choose to focus on low SEM data regimes. Therefore, the target heuristic $\mathrm r_t = 0.5$, which demonstrates the lowest best FID $\mathrm \in [6,8]$, is chosen as our optimal target heuristic $\mathrm r_t$ for further experiments.

### How many SEM images do we need?

In Karras et al.^8^, it has been shown that the StyleGAN2 with ADA can generate high-quality images with datasets of size as small as 1336 $\mathrm 1024 \times 1024$ images from the MetFaces dataset, a custom collection of painted human faces extracted from the Metropolitan Museum of Art online collection^32^, or 1944 $\mathrm 512 \times 512$ images from the BreCaHAD dataset^33^, a collection of overlapping crops extracted from 162 breast cancer histopathology $\mathrm 1360 \times 1024$ images. This last dataset contains the closest type of data, i.e. set of microscopy images, to SEM images. For the MetFaces dataset, a transfer learning from the StyleGAN2 pre-trained on an FFHQ-140K (140000 samples of human faces) with matching resolution improves the kernel inception distance (KID)^34^, a second metric similar to the FID (the FID is not reported on transfer learning tasks in Karras et al.^8^), by $\mathrm \sim 66.4\%$ in comparison to learning from scratch with ADA. However, for the latter dataset, transfer learning alone degrades the KID by $\mathrm \sim 16.7\%$ or slightly improves it by $\mathrm \sim 33.7\%$ if additional freezing of the first ten layers of the discriminator (Freeze-D^35^) is applied. Contrary to what is suggested in Karras et al.^8^, the similarity between the dataset used for pre-training the StyleGAN2 and a much smaller dataset, on which a transfer learning is applied determines if a transfer learning is beneficial or not to the quality of generated images. In the case of the BreCaHAD dataset, as well as our dataset of SEM images, this dissimilarity with FFHQ-140k images is particularly striking. Textures dominate the formers, and the latter by calibrated shapes. The calibration comes from the placement of recognisable and shared characteristics such as nose, eyes, and mouse location, as well as the aspect ratio of the figures-a kind of calibration challenging to apply to textural data. Additionally, no large dataset with textural characteristics is now available. Therefore, how far can we stretch the capabilities of the StyleGAN2 for a small dataset of SEM images when trained with ADA from scratch?

**Figure 3:**
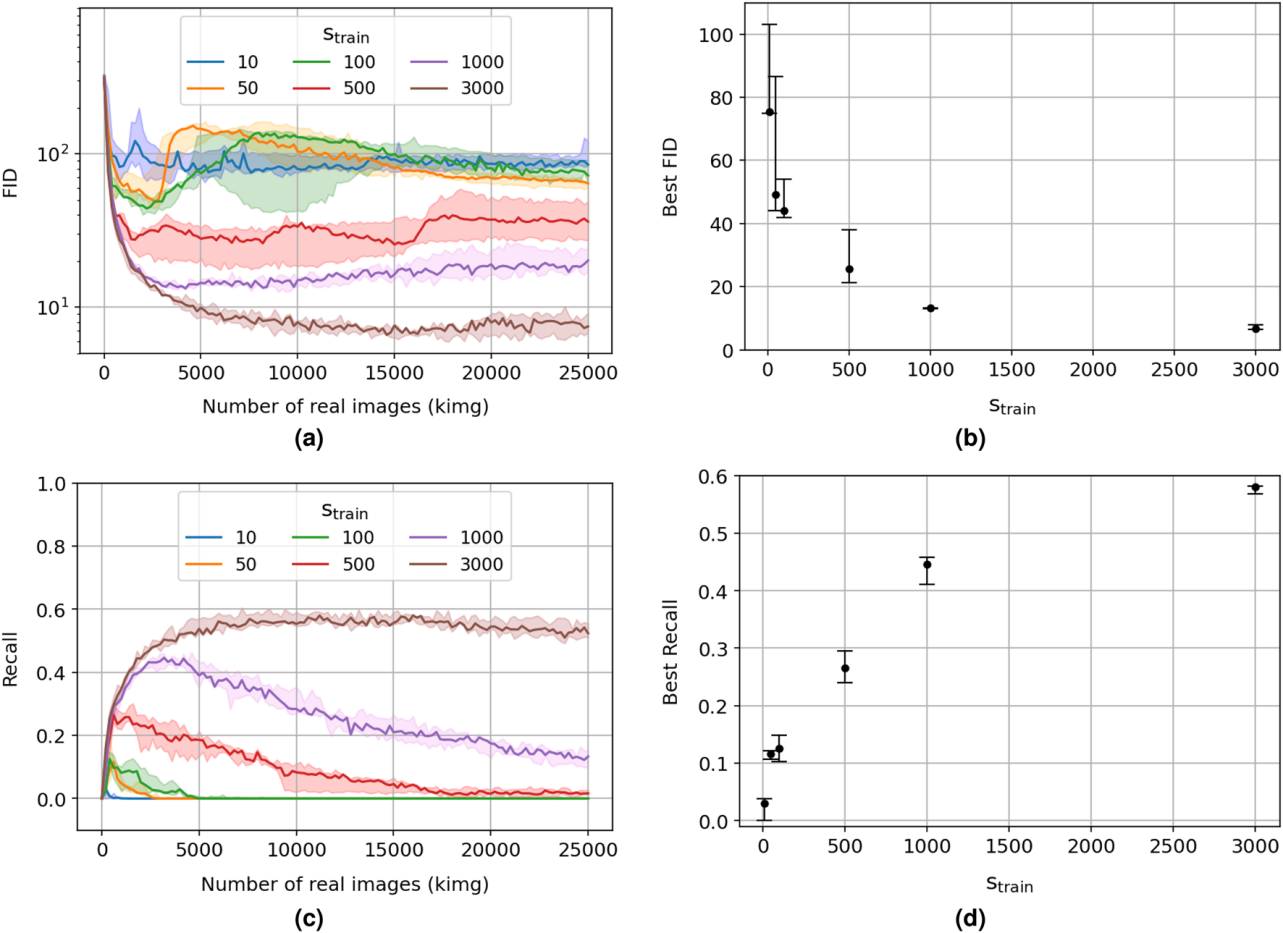
(**a**,**c**) Fréchet inception distance^36^ (FID, lower is better) and recall^37^ (higher is better) on generated SEM images as a function of the number of real images (per thousand images, kimg) presented to the discriminator during training of the StyleGAN2^8^ for different sizes of the training set: 10 (blue), 50 (orange), 100 (green), 500 (red), 1000 (violet), and all (brown) 3000 SEM images; (**b**, **d**) Corresponding median (black dot), minimum (lower cap), and maximum (higher cap) values for the best FID and recall are shown for the different sizes of the training set.

**Figure 4:**
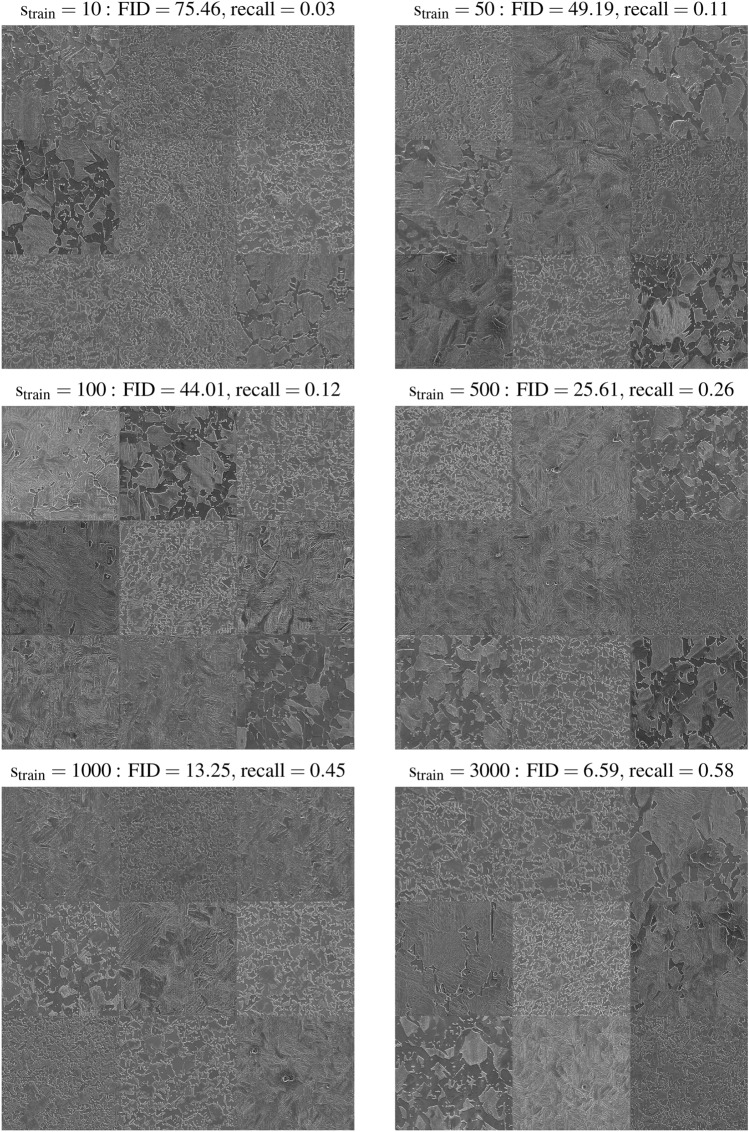
Samples of non-curated SEM images generated with the StyleGAN2 with ADA^8^ trained from scratch on a size of the training set, $\mathrm s_{train} = 10,\,50,\,100,\,500,\,1000,\,3000$ (all) SEM images from our dataset. For each $\mathrm s_{train}$, the best Fréchet inception distance^36^ (FID, lower is better) and recall^37^ (higher is better) median values are reported. Training runs are conducted with pixel blitting and geometrical transformations as ADA and a target heuristic $\mathrm r_t = 0.5$.

To answer, we train the StyleGAN2 with ADA over three consecutive runs with individual random seeds applying the pixel blitting and geometrical transformations as ADA and a target heuristic $\mathrm r_t = 0.5$, as reasonably stated above. Figure 3a and c respectively show the evolution of the convergence of the FID and recall^37^, a measure of the diversity of generated images (see Methods), as functions of the size of the training set, $\mathrm s_{train}$: 10 (blue), 50 (orange), 100 (green), 500 (red), 1000 (violet), and all (brown) 3000 available SEM images. It is worth noting that the ADA protocol is less essential at sustaining a constant level of recall over the entire training progress than from an increase in $\mathrm s_{train}$ compared to the FID. Therefore, the quality and diversity of generated SEM images should be improved through a bilateral search of a shared optimum in FID and recall. Then, the evolution of the FID over the training progress exhibits much more statistical stability once $\mathrm s_{train}$ reaches $\mathrm 1000$ kimg and above. Furthermore, Fig. 3b and d summarises the corresponding impact of the training set size on the best attainable FID and recall median value (black dots), respectively, with their corresponding minimum and maximum values (error bars). Overall, the best FID linearly drops with the logarithm of the training set size (see Fig. 2a in Supplementary Materials) when the best recall linearly improves with the square root of the training set size (see Fig. 2b in Supplementary Materials). This difference in the regime of improvement between FID and recall suggests that a further increase in the size of our dataset would benefit the diversity of generated SEM images than their already high quality. In terms of best FID and recall, we achieve $\mathrm{ FID = 13.25, 6.59}$ and $\mathrm{ recall = 0.45, 0.58}$ with a training set size of 1000 and 3000 SEM images, respectively, which performs better than for MetFaces and BreCaHAD datasets with $\mathrm{ FID = 18.22, 15.71}$ and $\mathrm{ recall = 0.35, 0.34}$, respectively. From a stability perspective, it is clear from Fig. 3a and c that the training of the StyleGAN2 with ADA becomes more reliable with a training dataset containing from 1000 SEM images, as one can see from the apparent differences in FID and recall among the three attempted individual runs. It is expected that a more extensive statistical study may reinforce this statement. Finally and from an aesthetic perspective, one can visually apprehend the evolution in diversity and quality of generated SEM images as a function of $\mathrm s_{train}$ in Fig. 4, with their reported best FID and recall. Among the presented eight generated samples per size $\mathrm s_{train}$, a bias towards the training set tends to vanish as $\mathrm s_{train}$ increases, and the quality of generated samples becomes visually appealing passed $\mathrm s_{train} = 500$ images. Generated SEM images reproduce the microstructural characteristics of ferrite-martensite DP steel. On a large scale, with lighter contrast, the martensite phase is dispersed in the form of islands surrounded by a matrix of ferrite phase, with darker contrast. The interface of martensite islands is irregular, as observed in the case of real ferrite-martensite DP steel microstructures. Moreover, on a smaller scale, lamellar substructures are well distinguishable within the martensite phase.

### Interpolation between dissimilar microstructures

It is common in the literature related to computer vision to visualise the interpolative behaviour of a generative model to assess the smoothness of the high-dimensional space of image representation emerging from the generator. In the present context, interpolation between generated samples highlights the possibility of predicting the look of a microstructure if synthesised with processes shared by two or more mismatched microstructures. Indeed, a first microstructure may possess thermal and mechanical properties, $\mathrm p_1$ and $\mathrm q_1$, a second microstructure the properties $\mathrm p_2$ and $\mathrm q_2$, with an existing trade-off between $\mathrm p$ and $\mathrm q$. Therefore, the interpolation among distinctive microstructures, with no necessarily overlapping properties of interest, may help experimentalists to foresee the type of alloy microstructure to synthesise for a target application.

**Figure 5:**
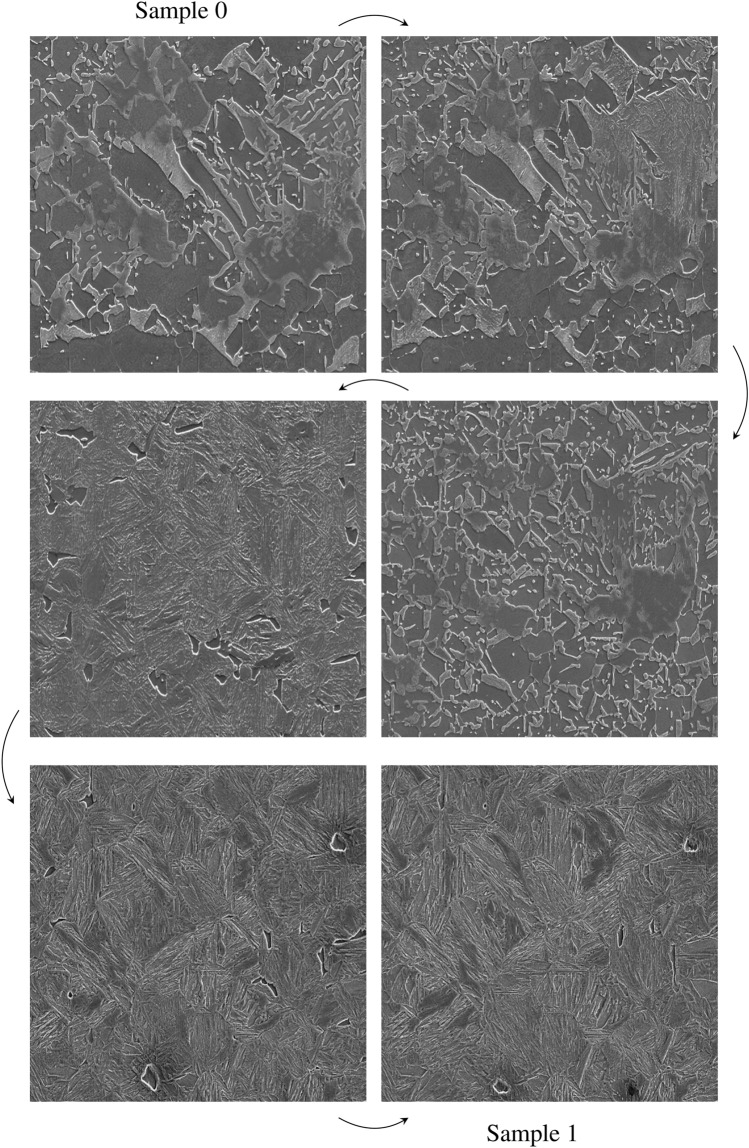
Selection of 4 non-curated generated SEM images obtained thanks to a spherical linear interpolation^38^ between sample 0 (top-left corner) and sample 1 (bottom-right corner), themselves generated from two random numerical vectors of 512 dimensions fed as input to the StyleGAN2 trained with ADA^8^ from scratch on 3000 SEM images. The direction of the sequential flow from one interpolated image to another is illustrated with an arrow. A one-minute smooth video of this sequence is available online (see Data availability).

To this end, we use a spherical linear interpolation (slerp), introduced by Shoemake^38^ for computer graphics, which is more suited than linear interpolation to traverse a high-dimensional latent space^39,40^. We first obtain two latent numerical vectors of 512 dimensions sampled from a standard normal distribution ${\mathscr {N}}(0,1)$ and then compute 1438 intermediate latent vectors according to the slerp between them. Next, the StyleGAN2 with ADA^8^, trained above on the 3000 SEM images available, is used for generating the related $\mathrm 512 \times 512$ SEM images. Figure 5 shows a sample of four SEM images out of this interpolative generation, where the starting (sample 0, top-left corner) and ending (sample 1, bottom-right corner) SEM images are added, and a corresponding one-minute high-quality video is made available online (see Data availability). This short sequential flow of intermediate microstructures shows the diversity of microstructural characteristics (e.g., size, shape, type, and fine-structure of grains and grain boundaries, gaps) that one can observe by traversing the high-dimensional latent space learnt by the StyleGAN2 with ADA from a relatively small dataset of SEM images. It is worth remarking on the high smoothness of the latent space learnt by the StyleGAN2 with ADA, such that small changes in the latent space result in slight perceptual modifications in the space of SEM images. It is recommended to watch the video shared online for a better appreciation (see Data availability).

## Discussion

In the present study, we show that the StyleGAN2 architecture trained with the ADA protocol is a viable tool for generating high-quality and diverse $\mathrm 512 \times 512$ SEM images of ferrite-martensite DP steel alloy in low data regime, with a mild change in the training strategy in comparison to its application to FFHQ images of human faces. Indeed, pixel blitting and geometrical transformations, with a target heuristic $\mathrm r_t = 0.5$, show evident effectiveness in stabilizing and reducing the FID along the training process with a data set size starting from $\mathrm 1000$ SEM images. Also, even though it is clear that the generation of compelling synthetic SEM images cannot replace the integration of actual SEM data, it has the potential to strengthen the role of SEM data in materials science.

Speich and Miller^41^ show that a relationship between martensite fraction, carbon content and ferrite grain size in DP steels with their tensile properties exists. Indeed, those above compositional and morphological features are observed to correlate with tensile and yield strengths but not ductility. For ductility, Speich and Miller^41^ suggest that some unobserved microstructural characteristics are necessary to develop comprehension, but their nature is still to be uncovered. Likewise, Park et al.^17^ focused on the expected relationship between the martensite phase distribution in DP steels and their tensile properties (strain hardening, ductile fracture). They noted from optical and SEM images that a chain-like networked structure of martensite surrounding ferrite grains might improve the strength-ductility balance. This morphological feature still needs to be quantified. In this sense, FEM simulations of the strength-ductility behaviour of DP steels have been conducted with the support of experimental and synthetic phase maps to identify factors and estimate their salience in influencing the strength-ductility balance. Such as, recently, Shiraiwa et al.^23^ performed forward and inverse analysis of the structure-property relationship in DP steels. First of all, they prepared a large number of synthetic phase distributions of ferrite and martensite thanks to a Gaussian random field (GRF) method^42^, chosen for quickly generating phase maps in comparison to point-based (e.g. Voronoi tessellation) or physics-based (e.g. phase-field) methods. Furthermore, they assumed that there were no more than two phases, no chemical heterogeneity in each phase and that each phase was composed of multiple crystal grains. Under these assumptions, 2D synthetic phase maps composed of two-color patterns were created and used to define the spatial distribution of ferrite and martensite phases. Additionally, anisotropic shapes with extreme geometries, such as uniform and lamellar distributions, and periodicity were introduced in the phase maps for broadening diversity via a 2D Gaussian distribution with variable horizontal and vertical variances, and a rotation angle corresponding to the elongation direction of the phase map, as well as a tilling for tuning periodicity. More straightforward approaches to the generation of synthetic images exist, like in Ishiyama et al.^25^, where two experimental images issued from two different periods of annealing are superimposed by randomly matching equivalent microstructural features. This method can conserve the quality and noise level retrieved in real images. However, it has the inconvenience of strongly biasing generated images to microstructural features encountered in observed samples, with smooth interpolation of microstructural features being unreachable. Later on, in Shiraiwa et al.^23^, a list of geometric, topological, covariogram, spatial correlation, and persistent homology descriptors are calculated from the synthetic 2D phase maps in an attempt to uncover unanticipated structure-property relationships with TS and EL, both evaluated from the stress-strain curve calculated from crystal plasticity FEM (CPFEM) simulations based on synthetic phase maps with the incorporation of a ductile damage model^43^. A consequent forward analysis, where a pair of Random Forest models predict TS and EL, showed that the volume fraction of martensite (as widely reported for DP steels), the first Betti number (a topological descriptor representing the number of holes in the martensite phase), and SC1 (the first principal component of the spatial correlation function) were individually significant at supporting a TS prediction according to the descriptors’ importance in promoting the predictions performance. Interestingly, a prediction of EL was reported to be a much more complicated endeavour with a poorer prediction performance, and no salient descriptors were found, therefore suggesting that the list of considered microstructural descriptors is incomplete and that synthetic 2D phase maps contain an insufficient amount of information on microstructural features of DP steels.

Let us retroactively summarize the issues as mentioned above:

(i) Lack of microstructural information into hand-crafted descriptors extracted from phase maps and supporting machine learning-based prediction of TS and EL.
(ii) Lack of microstructural features represented in synthetic 2D phase maps built from conventional generative processes such as the GRF method with uncorrelated Gaussian noise, anisotropic Gaussian distributions, and tilling add-ons.
(iii) Lack of a sufficiently large set of diverse and detailed SEM images for attempting to construct a phenomenological and quantifiable understanding of relationships linking coarse and fine microstructural features to TS and EL in DP steels.

Also, we propose that a StyleGAN2 trained with the ADA protocol presents all the essential characteristics for a generative process to respond to these issues. Its advantages are as follows:

(i’) A generative process like the StyleGAN2, once trained on a small dataset of SEM images ($\mathrm \gtrsim 1000$) with the ADA protocol, can virtually deliver an unlimited number of synthetic high-quality SEM images of a diversity principally constrained by the joint distribution of all the coarse and fine microstructural details existing in the original set of real SEM images used for training the generative process. In other words, all the combinations of microstructural features that could be observed from experimentally acquired SEM images of DP steels are theoretically accessible under various spatial arrangements, volume fractions, shapes, and heterogeneity of ferrite and martensite phases thanks to the generative process exposed in this study. A process of microstructural exploration that is not affordable with real SEM observations and not reproducible with conventional generative processes like the GRF method.
(ii’) It is expected that conventional generative processes like the GRF method are about to be supplanted by methods similar to the StyleGAN2 with ADA with the intent to serve high-quality synthetic 2D phase maps where fine microstructural details, like a lamellar martensite phase or martensite islands in ferrite, are reproduced and are expected to have an impact on the tensile properties of DP steels, pushing further forward studies such as in Briffod et al.^43^ and Shiraiwa et al.^23^. Furthermore, it is worth examining the case of an inverse design performed in Shiraiwa et al.^23^ where it consists in creating a wide variety of synthetic 2D phase maps in advance and search for the ones possessing the highest $\mathrm{TS} \times \mathrm {EL}$ calculated by CPFEM with a ductile damage model. Also, even if some synthetic 2D phase maps are identified as good candidates for improving $\mathrm{TS} \times \mathrm {EL}$, all of them cannot lead to DP steel microstructures which are consistent in morphology and grain details with real images, the spatial arrangement and the volume fraction of phases representing just a sub-group of coarse microstructural features, and none of the fine phase details. This is a clear bottleneck to the inverse design as informed by synthetic 2D phase maps from a GRF-like method that is to be overcome with the StyleGAN2 with ADA.
(iii’) In the forward and inverse analysis aforementioned, if this is about to inform machine learning models for predicting TS and EL or to support a Bayesian optimization for efficiently finding the best synthetic 2D phase maps with high $\mathrm{TS} \times \mathrm {EL}$, all of them are based on hard-coded microstructural descriptors which, according to Shiraiwa et al.^23^, cannot represent the whole set of characteristics embedded in SEM images. To this end, unconditional generative models like the StyleGAN2 are self-supervised learners. It can isolate the whole microstructural features from SEM images without human supervision and compress them into high-dimensional numerical vectors, indeed its 512D input vectors. For a StyleGAN2, an infinitesimal transformation of its input vector will impact the consequent generated SEM image depending on where the transformation is performed on the input vector. Thus, high-dimensional input, or feature, vectors are proxies to the SEM images that encapsulate all the microstructural features of SEM images from coarse to fine details of phases. They and their derivatives can serve machine learning models to predict TS and EL and help clarify complex microstructure-property relationships. Indeed, following the closed-form factorization latent semantics in GAN proposed by Shen and Zhou^44^, primary factors of variation supporting the differentiation among SEM images can be extracted, and *a posteriori* used as inputs to supervised machine learning methods for regression on physical attributes (e.g. thermal or mechanical properties), or for semantic segmentation of SEM images (e.g. autonomous extraction of phases, grain boundaries). Additionally, The inverse design of microstructures that possess target properties, which potentially obey a mutual trade-off, for instance, the strength-ductility $\mathrm{TS} \times \mathrm {EL}$ trade-off encountered in DP steels, for serving a given purpose. For this third application, a general tendency to find an imbalance between unlabeled and labelled data limits the use of conditional generative models, where the association of a latent vector and a set of material properties would be used as inputs to generate an image. The joint association of an unsupervised generative model and a supervised regressive model would then have to be employed for attempting an inverse design.

More broadly, the main ambition of this study is to trigger the use of generative models on SEM images and to serve as a guideline for future improvements. First, the various types of augmentation used for ADA must be meaningful to the field of images it applies. For example, median or Gaussian blur filtering^45^ are among the candidate transformation that may help more in stabilizing the training process of the StyleGAN2 through ADA than image-space filtering, additive noise, or color-based transformations. Second, the ADA protocol is sub-optimal because the FID (quality) and recall (diversity) metrics are unequally impacted. In this sense, the choice of an optimal target heuristic $\mathrm r_t$ value, which simultaneously assesses the FID and recall metrics, may be attempted. Finally, and as already emphasized in Karras et al.^8^, the styleGAN2 may deserve a specific fine-tuning of its architecture for increasing the potential of ADA on smaller datasets of SEM images ($\mathrm \ll 1000$ images).

## Methods

### Protocols of synthesis and observation of ferrite-martensite dual-phase steel sheets

**Figure 6:**
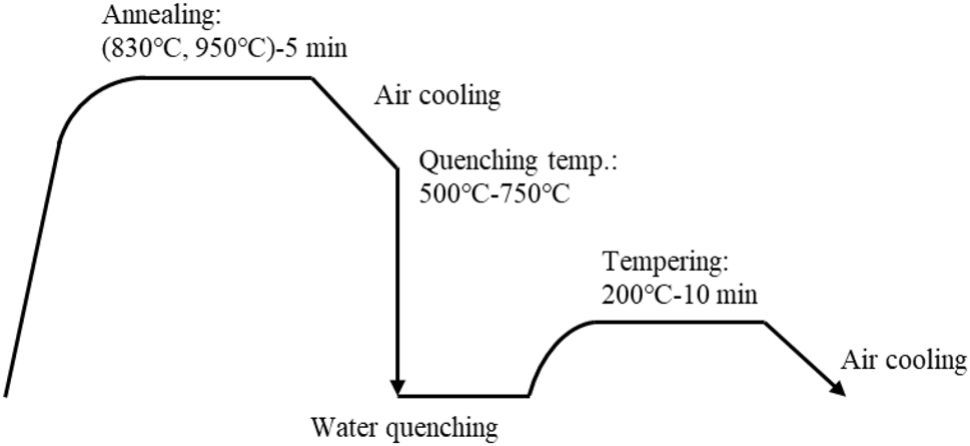
The sequence of heat treatments applied to cold-rolled Fe-C-Si-Mn steel sheets of 1.4 mm in thickness for obtaining ferrite-martensite dual-phase (DP) steel sheets.

Fe-C-Si-Mn steels were smelted in a 150 kg vacuum electric furnace and cast into moulds to form steel ingots. The Si and Mn contents were fixed to 2.0 wt%, and the C contents were changed to 0.10 wt%, 0.15 wt%, and 0.20 wt%. Next, the steel ingots were hot rolled to form hot-rolled steel sheets with a sheet thickness of 3.8 mm, followed by both sides grinding to a sheet thickness of 2.8 mm. After that, cold rolling with a reduction ratio of 50% was performed to obtain cold-rolled steel sheets having a thickness of 1.4 mm. Next, the cold-rolled steel sheets were subjected to the heat treatment shown in Fig. 6 to obtain ferrite-martensite dual-phase (DP) steel sheets. During the heat treatment, the fraction of martensite was adjusted by tuning the annealing and quenching temperatures. As a result, a set of 30 kinds of DP steel sheet was prepared with a martensite fraction ranging from 37% to 100%. For the observation of the DP steel sheet microstructures via scanning electron microscopy (SEM), specimens were prepared by sectioning parallel to the rolling direction. Then, a mechanical polishing and a subsequent etching in a 3 vol.% nital solution were applied. The SEM images were taken using a Schottky field emission scanning electron microscope SU5000 (Hitachi High-Technologies) with an acceleration voltage of 15 kV, a working distance of 5 mm, and a magnification of 1000. A set of 100 SEM images was obtained per sample, bringing a total of 3000 SEM images of ferrite-martensite DP steel sheet microstructures.

### Preliminary processing pipeline on the SEM data

The preliminary processing of the acquired SEM images in the dataset follows a pipeline to normalise their format, taking conditions (e.g., exposure, sharpness), and size. To this end, SEM images are first standardised to an 8-bit grayscale portable network graphics (PNG) format. Then, we adjust images by histogram matching so that their cumulative histogram, showing the number of pixels for every 256 shades of grey, matches that of a reference chosen for its uniform contrast, sharpness, and noiseless characteristics. Next, a prior median filter on source images and the histogram matching are performed thanks to the scikit-image Python library^45^. Finally, all SEM images are centred cropped to a size of $\mathrm 512 \times 512$ images. These last constitute our dataset of SEM images for training the StyleGAN2 with ADA^8^.

### StyleGAN2 architecture

In this paper, we use the architecture StyleGAN2^8^ for the generative adversarial network (GAN). We keep unchanged most of the implementations details of the official PyTorch version^31,46^, except for the training and generation steps, as well as for the type of processed transformations during ADA, where slight modifications and amendments are performed. For example, for the training step, we include the option to perform a run on multiple graphical processing units (GPU), starting from a first GPU with a given identification number for setting up multiple runs in parallel on a machine. This is particularly useful when studying the dependency in performance of the StyleGAN2 on the type of used transformations during ADA or the target heuristic $\mathrm r_t$ (see Results). Furthermore, in the list of transformations available during ADA, we voluntarily switch off the Luma flip from the color-based transformations to check its impact on training stability (see Supplementary Materials). Finally, we replace the official generation pipeline^31^ with the one shared by D. Schultz^47^ for performing spherical linear interpolation^38^ between SEM images (see Results).

All the training runs of the StyleGAN2 with ADA were performed on 4 GPUs in parallel out of 16 Tesla V100 SXM3-32GB GPUs from a single NVIDIA DGX-2, using PyTorch (torch 1.7.1+cu101, torchvision 0.8.2+cu101), CUDA 10.1 (Update 2), and cuDNN 8.0.5. Each training run was stopped once the discriminator had seen 25M real SEM images. The mini-batch size was computed according to the official implementation^31^ to keep the memory consumption of GPUs low. For each study performed in Results on the dependency of the FID and recall metrics on the type of augmentation, target heuristic $\mathrm r_t$, and on the size of the training set, three consecutive training runs with individual random seed are performed, the hardware and time limitations constraining the analysis to a low statistical regime. Overall, the project consumed a total of $\sim 17\,\mathrm{MWh}$ of electricity.

### Fréchet inception distance

An arbitrary image can be represented by a vector of $\mathrm d=2048$ dimensions issued from the latent representational space of an InceptionV3 classifier^48^. Thus, two sets $\mathrm p$ and $\mathrm q$ of real and generated images, respectively, can be defined as two d-dimensional multivariate Gaussian distributions, $\mathrm p = {\mathscr {N}}(\mu _{p},\Sigma _{p})$ and $\mathrm q = {\mathscr {N}}(\mu _{q},\Sigma _{q})$, of mean $\mathrm \mu$ and covariance matrix $\mathrm \Sigma$. Also, we compute as in Karras et al.^8^ the Fréchet inception distance (FID)^36^, or Fréchet distance^49^, to measure the similarity between the two distributions $\mathrm p$ and $\mathrm q$, as the FID shows high consistency in the assessment of image quality and similarity (see appendix A1 in Heusel et al.^36^).

More precisely, the FID is a 2-Wasserstein distance between the two probability measures $\mathrm p$ and $\mathrm q$ on $\mathrm {\mathbb {R}}^{d}$. The FID(p,q), or $\mathrm W_{2}(p,q)$, is expressed as^50^:

1 $$\begin{aligned}\textrm{FID}(p,q) = \left\Vert \mu _{\textrm{p}} - \mu _{\textrm{q}} \right\Vert _{2}^{2} + {\textrm{tr}}(\Sigma _{\textrm{p}} + \Sigma {\textrm{q}} - 2(\Sigma _{\textrm{p}}\Sigma _{\textrm{q}})^{1/2}) \, , \end{aligned}$$

where $\left\Vert . \right\Vert _{2}$ denotes the $\mathrm L^{2}$-norm, and $\mathrm tr({\mathscr {M}})$ the trace of a matrix $\mathrm {\mathscr {M}}$. Lower is the FID, closer are the two distributions $\mathrm p$ and $\mathrm q$, and more similar are real and generated images. Regardless of the size of the training set, we compute the FID between 50k generated images and all available training images, as recommended by Heusel et al.^36^.

### Recall metric

Following Kynkäänniemi et al.^37^, we compute a recall metric using *k*-nearest neighbours for evaluating the diversity of generated SEM images.

A representational, or feature, vector $\mathrm \phi$ of $\mathrm d=4096$ dimensions of an arbitrary image is extracted after the second fully connected layer of a pre-trained VGG-16 classifier^48^. Similarly to the FID, we draw 50k real and generated SEM images to form corresponding sets of representational vectors $\mathrm {\varvec{\Phi _{r}}} :=\left\{ \phi _{r} \in {\mathbb {R}}^{d} \right\}$, and $\mathrm {\varvec{\Phi _{g}}} :=\left\{ \phi _{g} \in {\mathbb {R}}^{d} \right\}$, respectively. Then, the recall, recall($\mathrm {\varvec{\Phi _{r}}}$,$\mathrm {\varvec{\Phi _{g}}}$), is defined as the proportion of real images located within an estimated manifold of generated images such as:

2 $$\begin{aligned}\textrm{recall} \left( {\varvec{\Phi _{r}}},{\varvec{\Phi _{g}}} \right) = \frac{1}{|{\varvec{\Phi _{r}}}|} \sum _{\phi = \phi _{r}\in {\varvec{\Phi _{r}}}} {\textrm{f}}\left( \phi ,{\varvec{\Phi _{g}}}\right) \,, \end{aligned}$$

where $\mathrm |{\varvec{\Phi _{r}}}|$ is the number of real images (50k here) composing the set $\mathrm {\varvec{\Phi _{r}}}$, and $\mathrm f\left( \phi ,{\varvec{\Phi _{g}}}\right)$ is a binary function such that:

3 $$\begin{aligned}{\textrm{f}}\left( \phi ,{\varvec{\Phi _{g}}}\right) = {\left\{ \begin{array}{ll} 1, &{} \text {if} \, \Vert \phi - \phi '\Vert _{2} \le \Vert \phi ' - NN_{k}\left( \phi ',{\varvec{\Phi _{g}}} \right) \Vert _{2} \, \text {for at least one} \, \phi '\in {\varvec{\Phi _{g}}} \\ 0, &{} \text {otherwise} \, , \end{array}\right. } \end{aligned}$$

where $\mathrm NN_{k}\left( \phi ',{\varvec{\Phi _{g}}} \right)$ is the $\mathrm k^{th}$ nearest representational vector to $\mathrm \phi '$ issued from ${\varvec{\Phi _{g}}}$, and $\mathrm \Vert .\Vert$ denotes the $\mathrm L_{2}$-norm. We adopt $k=3$ for avoiding saturation of the recall, as empirically observed in Kynkäänniemi et al.^37^.

### Target heuristic $\mathrm r_t$

As defined under the adaptive discriminator augmentation (ADA) process in Karras et al.^8^, a probability $\mathrm p \in \left[ 0,1\right]$ of augmenting a mini-batch of images during the training process of the StyleGAN2 is adjusted every four mini-batches according to a heuristic threshold chosen to indicate an over-fitting on training data, with $\mathrm p = 0$ at the start. An increase or decrease in $\mathrm p$ reduces or encourages an over-fitting, respectively. The amount by which $\mathrm p$ is adjusted is chosen in Karras et al.^8^ such that $\mathrm p$ can quickly rise from 0 to 1 in 500k images as seen by the discriminator. The heuristic threshold, $\mathrm r_t$, is defined as follows:

4 $${\text{r}}_{{\text{t}}} {{ = }}\mathbb{E}{\text{[sign (D}}_{{{\text{train}}}} {\text{)] ,}}$$

where $\mathrm D_{train}$ denotes the discriminator outputs (a binary classification of images as real or generated), the $\mathrm sign$ function enforces the stability of $\mathrm r_t$^8^, and the expectation $\mathop {\mathrm {\mathbb {E}}}[.]$ is taken over four successive mini-batches (i.e. 256 images). In the present study, we target the $\mathrm r_t$ to respect a value of $\mathrm 0.5$ along the training process after analyzing the influence of this choice on the best achievable FID (see the Results section). If $\mathrm r_t$ passes over or under this threshold, the StyleGAN2 is considered to overfit or not on the training set, and the augmentation probability $\mathrm p$ is adjusted accordingly. For all the types of image transformation, the same values of target heuristic $\mathrm r_t$ and consequently tuned $\mathrm p$ are used.

## Supplementary Information

Supplementary Information.

## Data Availability

A one-minute video displaying a sequential flow of generated SEM images of ferrite-martensite DP steel sheet microstructures is available at the following URL: 10.6084/m9.figshare.20845306.v1. Overall, the data supporting this study’s findings are available upon reasonable request from the corresponding author.

## Code availibility

Codes used in the present study are available on the GitHub platform at the following URL: https://github.com/GLambard/StyleGAN2_ADA_SEM.
