# Supplementary material for: Generation of highly realistic microstructural images of alloys from limited data with a style-based generative adversarial network
Source: Sci Rep. 2023 Jan 11;13:566. doi: 10.1038/s41598-023-27574-8 (PMC9834308; doi:10.1038/s41598-023-27574-8)
Supplement: Supplementary file 1 — Supplementary Information. [file 41598_2023_27574_MOESM1_ESM.pdf]

Supplementary Materials: Generation of highly realistic microstructural images of alloys from limited data with a style-based generative adversarial network

Guillaume Lambard[1,*], **Kazuhiko Yamazaki**[2], **and Masahiko Demura**[1]

[1]National Institute for Materials Science (NIMS), Research and Services Division of Materials Data and Integrated System (MaDIS), 305-0044, Namiki 1-1, Tsukuba, Ibaraki, Japan
[2]JFE Steel Corporation, Steel Research Laboratory, 210-0855, 1-1 Minamiwataridacho, Kawasaki, Kanagawa, Japan
[*]LAMBARD.Guillaume@nims.go.jp

Inefficiency of a Luma flip transformation

Figure 1 shows the distribution of the FID as a function of the number of real SEM images (25000 kimg maximum) shown to the discriminator during the training of a StyleGAN2[1]. It can be seen that the application of a Luma flip (red, see Table 1(a,c-e) in Supplementary Materials for a visual appreciation of a color-based transformation with the Luma flip activated) highly contributes to the instability and inefficiency of the training process than without (blue). Therefore, the color-based transformation is reduced to the set {contrast, brightness} in Results. Hue rotation and saturation are two transformations that do not apply to single channeled greyscale images.

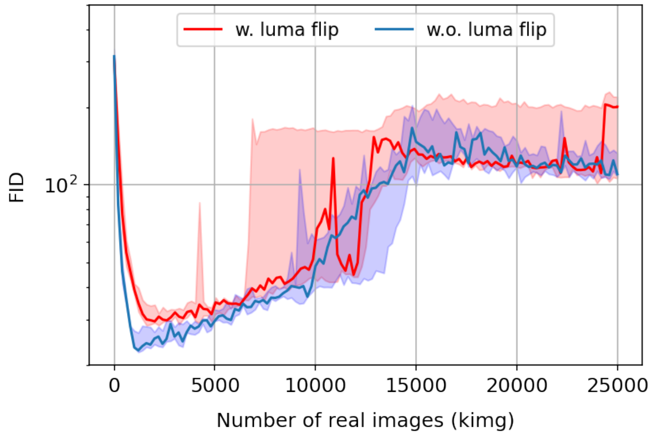

Figure 1. Fréchet inception distance (FID, lower is better) of generated to real SEM images as a function of the number of real images (per thousand images, kimg) shown to the discriminator during the training of a StyleGAN2[1] with application of a Luma flip (red) or without (blue). Solid lines and shallow areas indicate the median values as well as the minimum and maximum values, respectively, issued from three individual training runs.

Impact of available transformations

Type of transformations	a	b	c	d	e
Pixel blitting	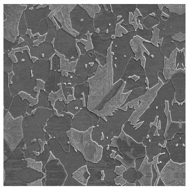	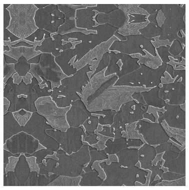	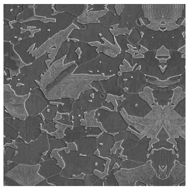	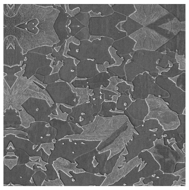	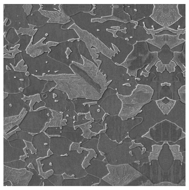
Geometrical	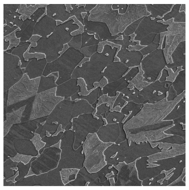	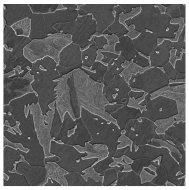	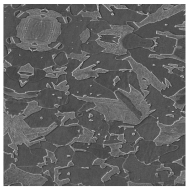	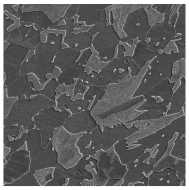	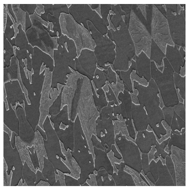
Color	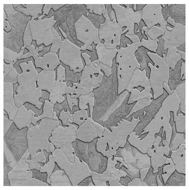	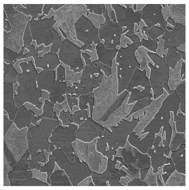	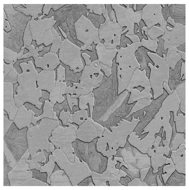	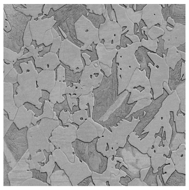	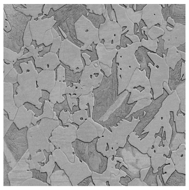
Image-space filtering	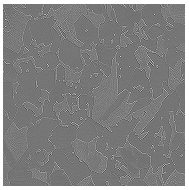	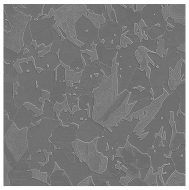	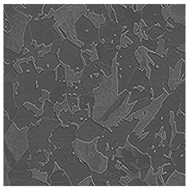	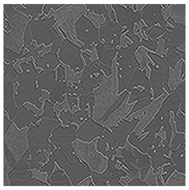	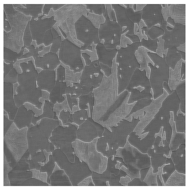
Additive noise	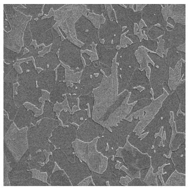	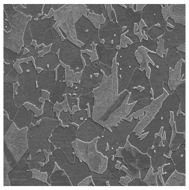	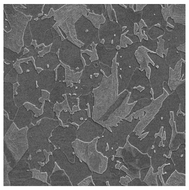	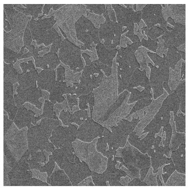	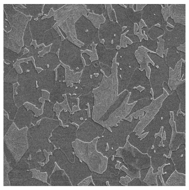
Cutout	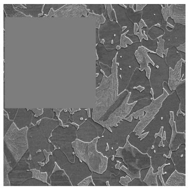	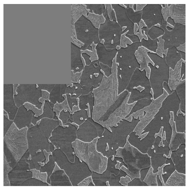	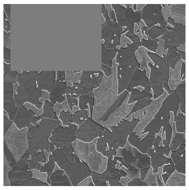	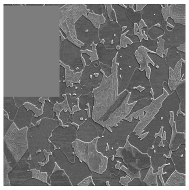	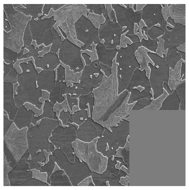

Table 1. Random selection of five examples (a, b, c, d, and e) of manipulation of an SEM image with pixel blitting (x-flip, 90° rotation, integer translation), geometrical (isotropic and anisotropic scaling, arbitrary rotation, fractional translation), color (brightness, contrast, Luma flip, Hue rotation and saturation (irrelevant to black and white images)), image-space filtering (over 4 distinct frequency bands), additive noise, or cutout transformation (see Karras *et al.*[1], pages 21-24 for details).

FID and recall as a function of the dataset size

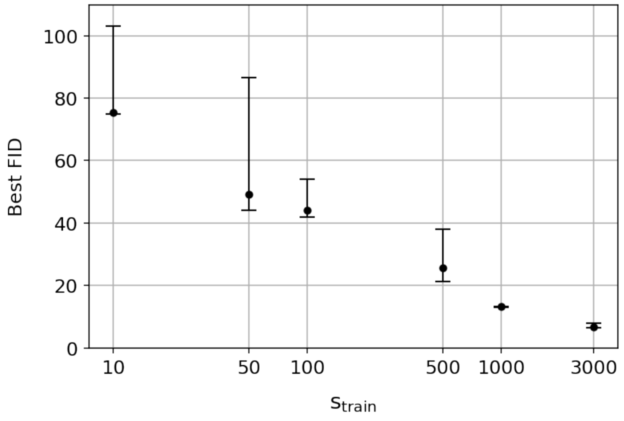

(a)

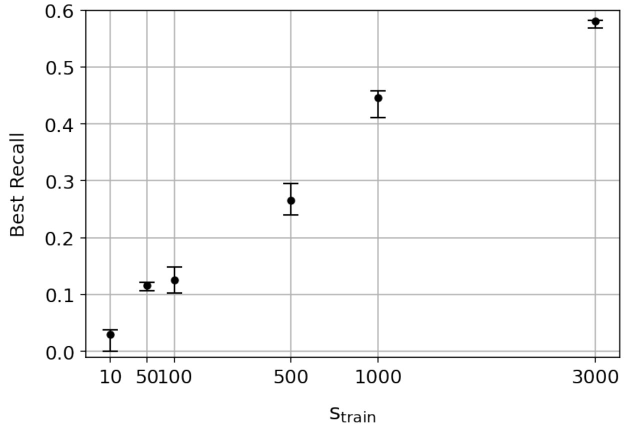

(b)

Figure 2. (a,b) Best median Fréchet inception distance[2] (FID, lower is better), and recall[3] (higher is better) values (black dots) on generated SEM images, with their corresponding minimum and maximum values (asymmetric error bars), as a function of the training set size: 10, 50, 100, 500, 1000, and all 3000 SEM images from our dataset. Values are obtained over three consecutive runs with individual random seed of training the StyleGAN2 with ADA[1] for 25000 kimg shown to the discriminator (see Figures 3a and 3c in the main text). A logarithm and square-root based scale are here used for highlighting the dependency of the FID and recall on the training set size, respectively.

Mosaic of real SEM images

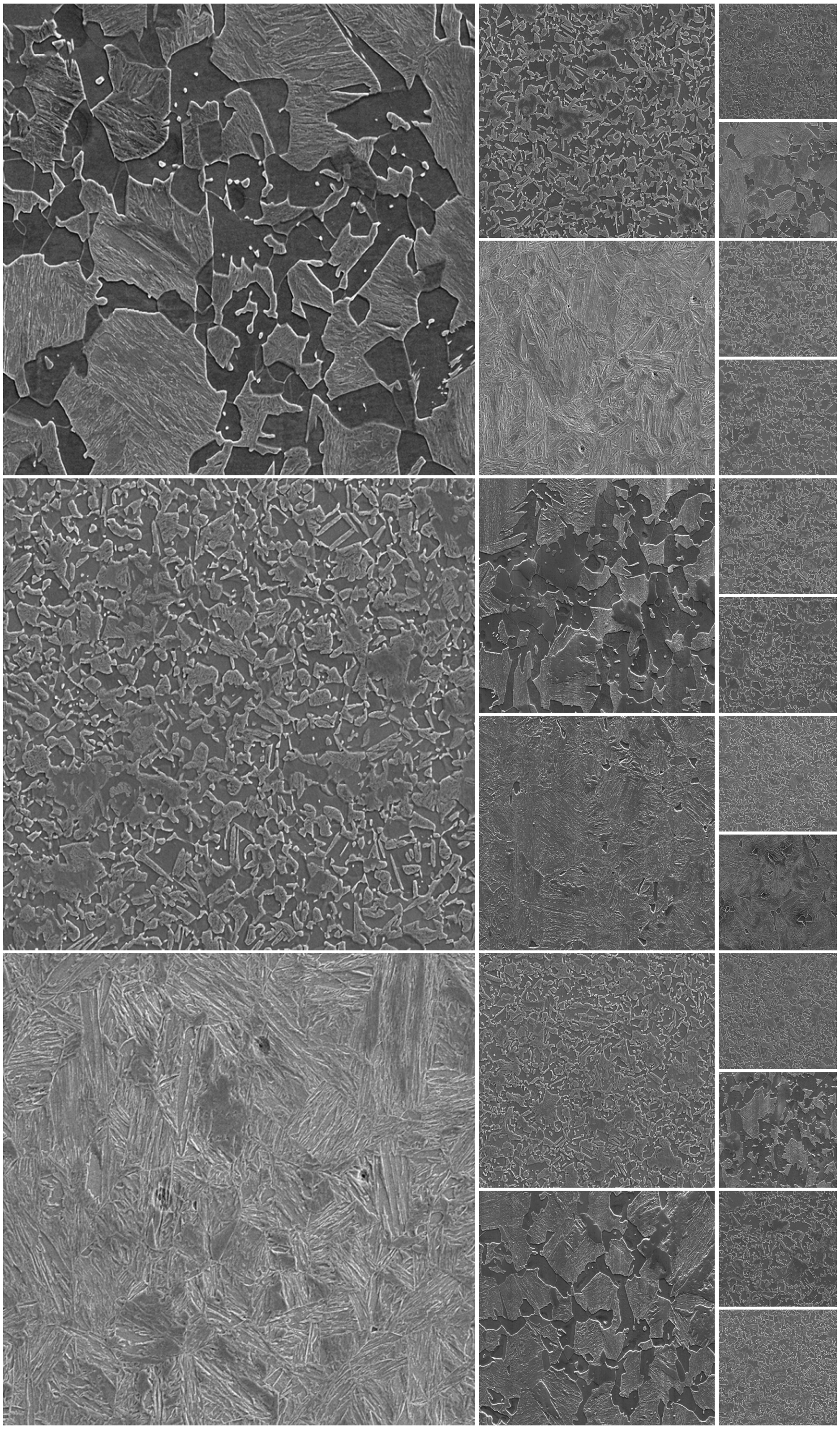

Figure 3. Random sample of 512×512 SEM images extracted from the initial dataset used in the present study. All the images are pre-processed following the pipeline detailed in Methods.

Mosaic of generated SEM images

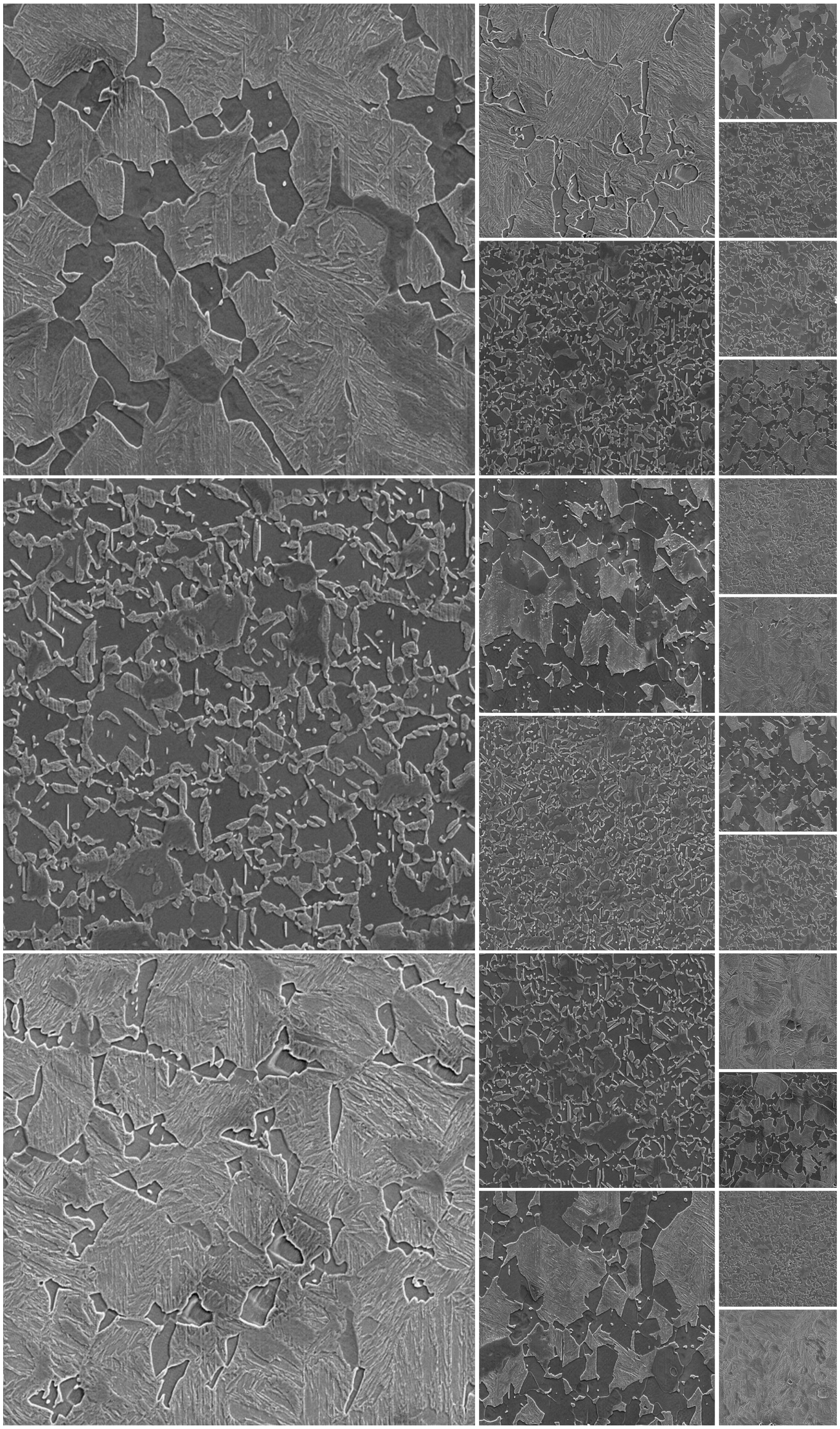

Figure 4. Random sample of non-curated 512×512 SEM images generated thanks to the StyleGAN2 with ADA[1]. It is recommended to zoom in for inspecting the fine microstructural characteristics in details.

References

1. Karras, T. *et al.* Training generative adversarial networks with limited data. In *Proc. NeurIPS* (2020).

2. Heusel, M., Ramsauer, H., Unterthiner, T., Nessler, B. & Hochreiter, S. Gans trained by a two time-scale update rule converge to a local nash equilibrium. In Guyon, I. *et al.* (eds.) *Advances in Neural Information Processing Systems*, vol. 30 (Curran Associates, Inc., 2017).

3. Kynkäänniemi, T., Karras, T., Laine, S., Lehtinen, J. & Aila, T. Improved precision and recall metric for assessing generative models. *Adv. Neural Inf. Process. Syst.* **32** (2019).
